# Functional Identification of Porcine *DLK1* during Muscle Development

**DOI:** 10.3390/ani12121523

**Published:** 2022-06-11

**Authors:** Yu Fu, Xin Hao, Peng Shang, Yangzom Chamba, Bo Zhang, Hao Zhang

**Affiliations:** 1 National Engineering Laboratory for Animal Breeding, College of Animal Science and Technology, China Agricultural University, Beijing 100193, China; b20173040294@cau.edu.cn (Y.F.); haoxin0331@cau.edu.cn (X.H.); 2 College of Animal Science, Tibet Agriculture and Animal Husbandry University, Linzhi 860000, China; shangpeng1984@xza.edu.cn (P.S.); yeyourong@xza.edu.cn (Y.C.)

**Keywords:** porcine, delta-like 1 homolog, muscle growth and development, Notch signaling pathway

## Abstract

**Simple Summary:**

Skeletal muscle is the largest tissue and serves as a protein reservoir and energy reservoir in the human and animal body. It also serves as the main metabolic activity site. The formation of skeletal muscle mainly depends on the differentiation and fusion of myocytes and other complex ordered processes; each step is regulated by various factors. In this study, we investigated the expression profiles, functional identification, and regulatory pathways of Delta-like 1 homolog (*DLK1*) in pigs and myocytes. We found that *DLK1* was highly expressed in the muscle tissues of pigs. *DLK1* promoted myocyte proliferation, migration, differentiation, fusion, and muscular hypertrophy, but suppressed muscle degradation. *DLK1* also inhibited the Notch signaling pathway by regulating the expression of key factors in the pathway, thereby producing a phenotype in which *DLK1* promotes muscle development. These findings provide valuable information to improve our understanding of the functional mechanisms of *DLK1* that underly myogenesis to accelerate the process of animal genetic improvement.

**Abstract:**

*DLK1* is paternally expressed and is involved in metabolism switching, stem cell maintenance, cell proliferation, and differentiation. Porcine *DLK1* was identified in our previous study as a candidate gene that regulates muscle development. In the present study, we characterized *DLK1* expression in pigs, and the results showed that *DLK1* was highly expressed in the muscles of pigs. In-vitro cellular tests showed that *DLK1* promoted myoblast proliferation, migration, and muscular hypertrophy, and at the same time inhibited muscle degradation. The expression of myogenic and fusion markers and the formation of multinucleated myotubes were both upregulated in myoblasts with *DLK1* overexpression. *DLK1* levels in cultured myocytes were negatively correlated with the expression of key factors in the Notch pathway, suggesting that the suppression of Notch signaling pathways may mediate these processes. Collectively, our results suggest a biological function of *DLK1* as an enhancer of muscle development by the inhibition of Notch pathways.

## 1. Introduction

Skeletal muscle development and growth are complex processes regulated by various factor networks [1,2,3], such as fibroblast growth factor [4], ferulic acid [5], signaling pathways [6], amino acids, and insulin-like growth factors [7]. Myogenesis includes a series of morphological changes from the embryonic stage and involves the proliferation, migration, differentiation, and fusion of muscle cells [8,9]. Embryonic myogenesis is essential for muscle fiber formation, whereas postnatal muscle growth mostly results from fiber hypertrophy [10]. Myofiber types and the number of satellite cell progenitors are also different throughout the developmental stages [11]. The meat production performance of agricultural animals is mainly dependent on muscle growth and development, and insight into the factors that regulate muscle development is crucial for treating muscle diseases. Therefore, it is important to study the development of skeletal muscle. 

*DLK1*, also known as preadipocyte factor 1 (Pref-1), was first discovered in neuroblastoma because of its inhibitory effect on preadipocyte differentiation [12]. DLK1 is a transmembrane glycoprotein with six tandem repeat epidermal growth factor (EGF)-like extracellular motifs [13]. *DLK1* is expressed in developing myofibers, associated satellite cells, and various tumors [14,15]. It is also widely expressed in various tissues during embryogenesis [16], whereas its expression is ceased in adult muscles and becomes restricted to neuroendocrine tissues and preadipocytes in both humans and mice [17,18]. The expression level of porcine *DLK1* in the embryo and during a short period after birth is significantly higher than that in other periods [19,20]. *DLK1* mRNA is markedly enhanced in muscles from callipyge sheep at 120 days of gestation through to 12 weeks of age [21]. *DLK1* expression was significantly increased in hypertrophied muscles [22]. The *DLK1* level rises in the presence of different myopathies, such as muscular dystrophies, following intense exercise and injuries [14,23,24,25]. These changes of *DLK1* expression affect the fate of cell differentiation [26].

In the metabolism, *DLK1* regulates fat formation and cell differentiation [27,28]. *DLK1* suppresses adipocyte differentiation, and *DLK1* interference enhances adipogenesis, showing that *DLK1* may maintain the preadipose state [29]. Pregnancy serum *DLK1* concentrations are related to indices of insulin resistance and secretion [30]. Glucocorticoids reduce *DLK1* expression, resulting in increasing adipose differentiation [31]. Lee found that *DLK1*-transgenic mice with a substantial loss of adipose tissue exhibited decreased insulin sensitivity, glucose intolerance, and hypertriglyceridemia [32]. Studies have also indicated a significant increase in muscle mass and a decrease in fat deposition at the *DLK1* locus in pigs [33,34]. Furthermore, *DLK1*-knockout mice display skeletal deformity, growth retardation, and obesity [35]. Muscle-specific *DLK1*-deletion also resulted in reduced skeletal muscle mass due to a reduction in the number of myofibers and the expression of the *MyoD* and *Myh4* genes [36]. Additionally, *DLK1* overexpression enhanced the differentiation of cultured myoblasts [36]. Sheep *DLK1* gene-coding mice showed muscle hypertrophy and pathobolism [37]. *DLK1* might also be involved in muscle regeneration [25,38]. *DLK1* is a member of the family of EGF-like repeat-containing proteins that include Notch/Delta/Serrate, which regulate cell fate determination, differentiation, and adipose tissue homeostasis [39]. Notch signaling has been found to be a key regulator of stem cell self-renewal and myogenesis in normal skeletal muscle [40]; it inhibits myogenic differentiation by the suppression of *MyoD* expression [41,42].

Existing information on the *DLK1* gene focuses on adipose differentiation; however, the specific regulatory role and mechanism of *DLK1* (especially the porcine *DLK1* gene) in muscle development is still poorly defined. In this study, we compared *DLK1* expression in pigs with different growth rates, explored its effects on various cytological processes in myogenesis, and elucidated the potential role and regulation pathway of *DLK1* in muscle development. Our data provide a basis for further research on the molecular regulation of muscle development in agricultural animals, including pigs, and will accelerate the process of animal genetic improvement.

## 2. Materials and Methods

### 2.1. Animal Samples

All animal procedures were approved by the China Agricultural University Animal Care and Use Committee (permit number SKLAB-2012-04-07). Embryonic tissue samples were taken from sacrificed Tibetan (TP), Wujin (WJ), and Yorkshire (YY) pregnant sows 60 days after insemination. TP and WJ pigs show slow growth characteristics, and YY are fast-growing pigs. The *longissimus dorsi* (LD) muscle tissues were sampled from the 12th rib. All animals were raised at the Tibet Agriculture and Animal Husbandry College.

### 2.2. Cell Cultures and Reagents

The C2C12 myoblast cell line (Type Culture Collection of the Chinese Academy of Sciences, Shanghai, China) was cultured in Dulbecco’s modified Eagle’s medium (DMEM, Gibco, Grand Island, NY, USA) containing 10% fetal bovine serum (FBS, Gibco, Grand Island, NY, USA) and 1% penicillin-streptomycin; the cell line was differentiated in DMEM supplemented with 2% horse serum (Gibco, Grand Island, NY, USA). The medium was changed every alternate day. The incubation environment was set to 37 °C and 5% CO_2_. 

### 2.3. Vector Construction and Transfection

Porcine *DLK1* complementary DNA (cDNA) (GenBank accession number: NM_001048187.1) was amplified by polymerase chain reaction (PCR) (forward: AAGCTTATGACCGCGACCGCA. Reverse: CTCGAGGCTTAGATCTCCTCGTCCCC) and then cloned into the pCDH vector from our laboratory. C2C12 cells were transiently transfected with *DLK1* overexpression plasmid to investigate the effects of porcine *DLK1* on myoblasts. Transfection was performed with Lipofectamine 2000 (Invitrogen, Carlsbad, CA, USA) according to the manufacturer’s instructions.

### 2.4. RNA Extraction, cDNA Synthesis, and Expression Analysis

Total RNA from cells or tissues was extracted using TRIzol Reagent (Invitrogen, Carlsbad, CA, USA) according to the manufacturer’s instructions, and then reverse transcribed into cDNA using the Transcriptase Kit (TIANGEN, Beijing, China). For expression analysis, semi-quantitative real-time PCR (SqRT-PCR) was performed as previously described [43], and the PCR products were analyzed using 1% agarose gel electrophoresis. Quantitative real-time PCR (qRT-PCR) was carried out on a Bio-Rad PCR System using SYBR Green Master Mix (TIANGEN, Beijing, China) and gene-specific primers. *GAPDH* was used as an internal control. Fold changes in the indicated genes were analyzed using the 2^−ΔΔCT^ method [44]. Proliferation-positive and -negative marker genes were chosen as in previous studies [45,46,47]. Primer sequences are listed in Table S1.

### 2.5. Proliferation Assay

The proliferation of the control and overexpression groups at 0 h and 12 h was observed under a microscope (Leica, Heidelberg, Germany). Cell Counting Kit-8 (CCK8) and 5-ethynyl-2-deoxyuridine (EdU) assays were used to analyze cell proliferation, which were performed as previously described [43]. Briefly, control and transfected cells were incubated with 10% CCK8 (Beyotime Biotechnology, Shanghai, China) at 37 °C for 1 h in the dark, and the absorbance was measured at 450 nm to determine the proliferation ability. For EdU staining, cells were incubated with 50 mM EdU (Ribobio, Guangzhou, China) at 37 °C for 2 h. EdU-positive cells were analyzed in the different treatment groups with Image J software.

### 2.6. Migration Assay

Cell migration was tested using Transwell and wound healing assays. Transfected cells were seeded into the upper Transwell chamber (6.5 mm diameter, 8.0 µm pore size; Corning Inc., Corning, NY, USA) with serum-free medium, and a complete medium was added to the lower chamber. After 12 h of incubation, the migrated cells were stained with crystal violet and observed under a microscope (ZEISS, Jena, Germany). Cells were seeded in 6-well plates and transfected. A wound line was created across the surface of the plates using a sterile plastic tip. The wounded cells were removed using PBS (Gibco, Grand Island, NY, USA) and cultured in 2% serum DMEM for 24 h. Migrated cells were photographed using a microscope (ZEISS, Jena, Germany).

### 2.7. Immunofluorescent Staining

Cells were fixed in 4% paraformaldehyde after washing with PBS. Fixed cells were permeabilized with 0.3% TritonX-100 and blocked for 1 h. The cells were then incubated with mouse anti-myosin heavy chain antibody (cat. no. M4276; Sigma-Aldrich, St. Louis, Missouri, USA, 1:500) at 4 °C overnight. Finally, the cells were incubated with fluorescently labeled secondary antibodies (cat. no. A11032; Thermo Fisher Scientific, Wilmington, DE, USA, 1:400) for 1 h at room temperature (about 25 °C) and DAPI (4′,6-diamidino-2-phenylindole) for 5 min. Digital images were captured using a fluorescence microscope (Leica image analysis system, model Q500MC). The fusion index was measured by dividing the number of nuclei found within the myotubes by the total number of nuclei in each image [48].

### 2.8. Statistical Analysis

Results are expressed as the mean ± standard deviation (SD) of three independent technical replicates and biological experiments, respectively. Student’s *t*-test was used to determine statistical significance, with * *p* < 0.05. considered significant [49]. Values of p and n are listed in the figure legends.

## 3. Results

### 3.1. Expression of DLK1 in Tissues of Pig Embryos

**Figure 1:**
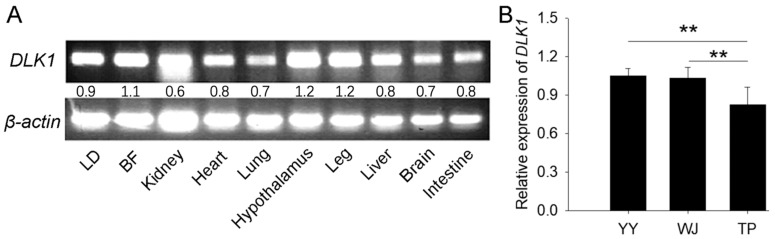
Expressions of *DLK1* in the embryonic tissues of pigs. (**A**) *DLK1* expression in the different tissues of TP pigs at the embryonic stage by SqRT-PCR. LD, *longissimus dorsi*; BF, back fat; (**B**) the mRNA expression levels of *DLK1* in the LD of three pig breeds. YY, Yorkshire (*n* = 6); WJ, Wujin pig (*n* = 6); TP, Tibetan pig (*n* = 6). Each bar represents the mean ± SD. ** *p* < 0.01.

*DLK1* was widely expressed in various tissues of pig embryos at 60 days post-insemination, a time that is in the middle of a crucial period of myofiber ontogenesis. The electrophoretogram displayed that its expression was much higher in the tissues of the *longissimus dorsi* muscle (LD), back fat (BF), hypothalamus, and leg muscle (Figure 1A). The expression of *DLK1* in the LD was lower in TP than in YY and WJ (Figure 1B), which was consistent with our previous transcriptomic results [50]. These results indicate that *DLK1* can regulate pig growth and development.

### 3.2. DLK1 Promotes Myoblast Proliferation

**Figure 2:**
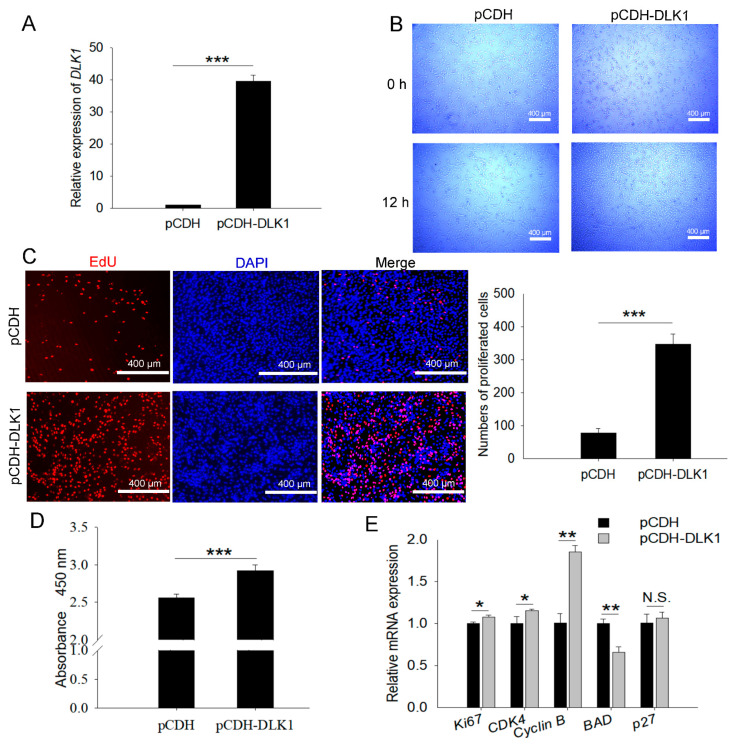
*DLK1* promotes myoblast proliferation. (**A**) Efficiency of the detection of plasmid overexpression; (**B**) microscopic view of cell proliferation; scale bar = 400 µm; (**C**) EdU staining for proliferated cells following pCDH-*DLK1* transfection. Nuclei are stained with DAPI; red indicates EdU-positive proliferating cells. Representative images are shown in the left panel, and the statistical graphs in the right panel indicate the proliferating cells 48 h after transfection; *n* = 3 in each group; scale bar = 400 µm; (**D**) CCK8 assay of proliferated myoblasts transfected with overexpression fragments; (**E**) the mRNA expression levels of proliferation marker genes. The data represent the mean *±* SD of three independent experiments. *GAPDH* was used as a reference gene. * *p* < 0.05, ** *p* < 0.01, *** *p* < 0.001, N.S. represents not significant.

The *DLK1* overexpression vector was constructed to investigate its biological functions in myoblasts (Figure 2A). Microscopic examination showed that the number of proliferating cells in the overexpression group was greater than that in the control group during the same growth period (Figure 2B). The EdU and CCK8 results showed that *DLK1* overexpression substantially improved EdU positivity compared with that of the control (Figure 2C), and it markedly increased the absorbance of cells after CCK8 treatment (Figure 2D). In addition, the expression of proliferation marker genes (*Ki67*, *CDK4*, and *Cyclin B*) was elevated, whereas proliferative inhibitors were decreased in C2C12 cells with *DLK1* overexpression (Figure 2E); this further increased the possibility that *DLK1* accelerates myoblast proliferation.

### 3.3. DLK1 Accelerates Myoblast Migration

**Figure 3:**
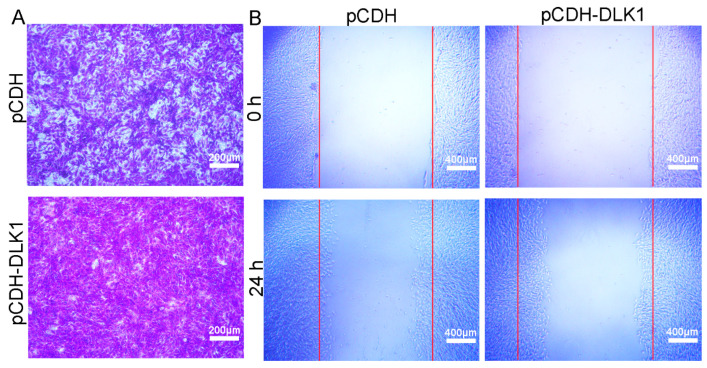
*DLK1* facilitates myoblast migration. (**A**) The effect of *DLK1* on cell migration was investigated using a Transwell migration assay. Purple represents migrated cells stained with 0.1% crystal violet; scale bar = 200 µm; (**B**) the wound-healing migration assay of C2C12 myoblasts. The red line represents the wound healing area; scale bar = 400 µm.

Transwell migration assays indicated that the number of migrated cells with *DLK1* overexpression was higher than that with the vector alone, suggesting that *DLK1* promoted the migration of C2C12 cells (Figure 3A). To confirm these results, wound healing assays were performed; C2C12/*DLK1* cells displayed a higher migration ability than C2C12/vector cells after 24 h (Figure 3B).

### 3.4. DLK1 Positively Regulates Myogenic Differentiation and Myogenin Expression

**Figure 4:**
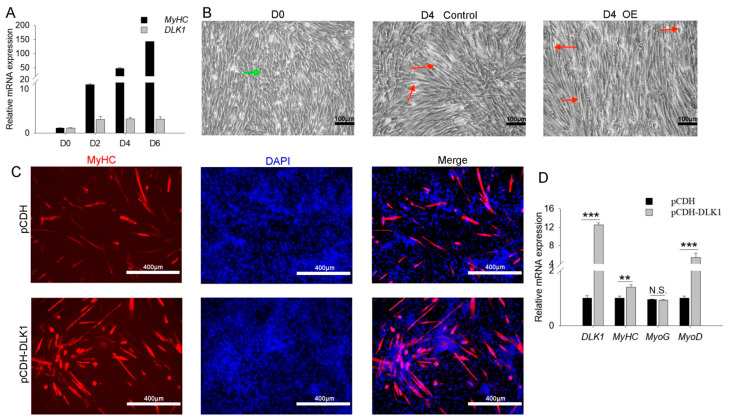
*DLK1* improved cell differentiation. (**A**) Quantitative real-time polymerase chain reaction (qRT-PCR) results showed the expression profiles of the *DLK1* gene during differentiation. *MyHC* is the myogenic-differentiation-indicator gene; (**B**) microscopic view of differentiated cells; D0 and D4 refer to differentiation after zero and four days. Control and OE represent cells transfected with pCDH and pCDH-*DLK1*, respectively; scale bar = 100 µm; green arrows represent undifferentiated myoblasts that displayed a disorderly and irregular morphology; red arrows represent orderly bundles of muscle tubes. (**C**) Immunofluorescence staining for MyHC protein in pCDH- or pCDH-*DLK1*-treated myoblasts that were cultured for four days in differentiation medium. MyHC and the nucleus are stained in red and blue (DAPI), respectively; scale bar = 400 µm; (**D**) the mRNA expression of *DLK1* and the differentiation marker genes, *MyoD*, *MyoG*, and *MyHC,* was quantified using qRT-PCR. ** *p* < 0.01, *** *p* < 0.001, N.S., not significant.

The time course of the changes in the myogenic and *DLK1* gene expression was detected in C2C12 myoblasts during differentiation. *DLK1* was elevated during the myogenic differentiation of C2C12 myoblasts, which was consistent with the changes in the expression levels of the myogenic marker, *MyHC* (Figure 4A). Microscopic views showed that *DLK1*-overexpressed myoblasts induced more orderly bundles of muscle tubes than the control during differentiation, and undifferentiated myoblasts displayed a disorderly and irregular morphology (Figure 4B). Immunocytochemical staining further confirmed that more myotubes were formed by myocytes following *DLK1* overexpression compared to control vector-transfected cells (Figure 4C). As expected, enforced *DLK1* expression in C2C12 cells dramatically enhanced the levels of myogenic markers (*MyHC* and *MyoD*), but *MyoG* expression was not significantly different (Figure 4D). Taken together, these results indicate that *DLK1* positively regulates myogenin transcription and myogenesis.

### 3.5. DLK1 Promotes Myogenic Fusion and Muscular Hypertrophy but Inhibits Muscle Degradation

**Figure 5:**
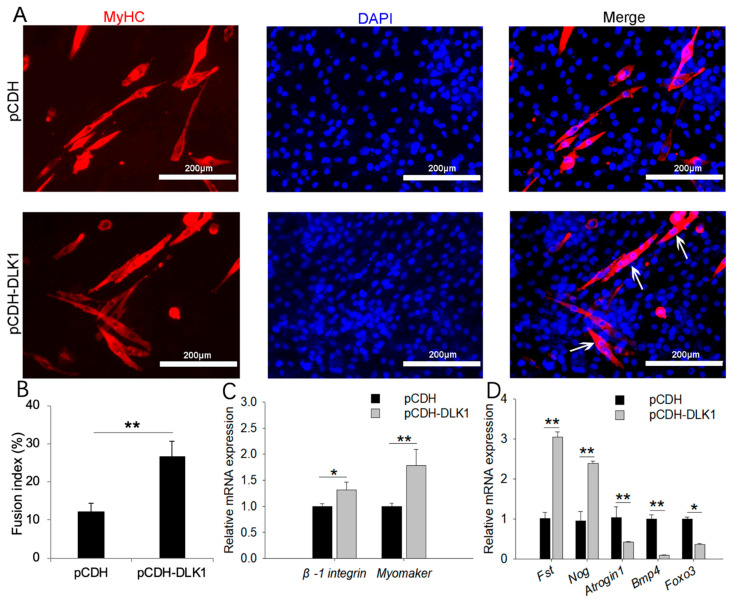
*DLK1* stimulated myoblast fusion and muscle hypertrophy but suppressed muscle degradation. (**A**) Myoblast fusion analysis by immunofluorescence staining for MyHC after four days of differentiation, and the white arrows represent the multinucleated myotubes; white scale bar = 200 µm; (**B**) the fusion index was measured by dividing the number of nuclei found within the myotubes by the total number of nuclei; (**C**) the mRNA expression of fusion marker genes was quantified by a quantitative real-time polymerase chain reaction (qRT-PCR); (**D**) the mRNA expression of muscle hypertrophy genes (*Fst* and *Nog*) and muscle degradation markers (*Atrogin1*, *Bmp4*, and *Foxo3*) was quantified by qRT-PCR. The data represent the mean *±* SD of three independent experiments. * *p* < 0.05, ** *p* < 0.01.

Myoblasts overexpressing *DLK1* displayed accelerated fusion kinetics, as demonstrated by the appearance of thick, large myotubes containing many myonuclei (Figure 5A,B). Consistent with the staining results, *DLK1* overexpression markedly increased the expression of fusion markers (*Myomaker* and *β-1integrin*) (Figure 5C). The *DLK1*-overexpressed treatment resulted in the significantly elevated expression of muscle hypertrophy genes, including *Fst* and *Nog*, whereas it downregulated the expression of muscle degradation markers (*Atrogin1*, *Bmp4*, and *Foxo3*) (Figure 5D).

### 3.6. Regulatory Pathway of DLK1 on Myogenesis

**Figure 6:**
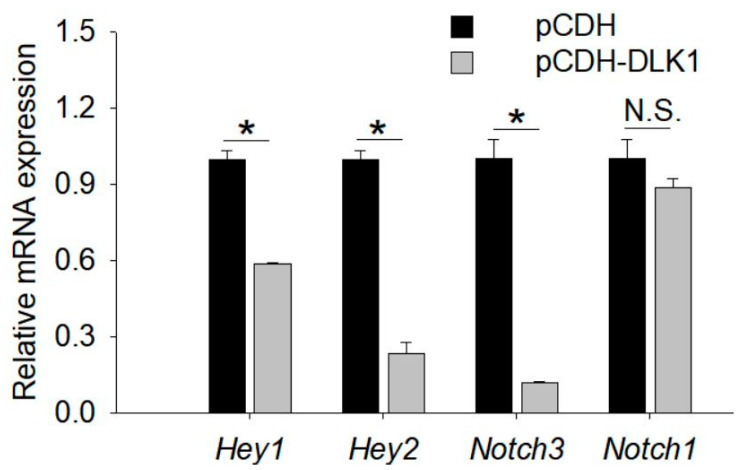
*DLK1* negatively regulated Notch signaling pathways. The mRNA expression of Notch-related genes in the control and *DLK1*-overexpressed myoblasts. The data represent the mean *±* SD of three independent experiments. * *p* < 0.05, N.S., not significant.

The Notch signaling pathway has been reported to be involved in muscle development [40]. Several genes related to the Notch signaling pathway were selected for validation by qRT-PCR (Figure 6). Notch-pathway-related genes (*Hey1*/*2* and *Notch3*) were prominently reduced at the mRNA level by *DLK1* overexpression in C2C12 cells. Among these Notch-related genes, only the expression of *Notch1* showed an insignificant downward trend with enhanced *DLK1* expression. These data indicate that *DLK1* may regulate muscle growth and development by inhibiting the Notch pathway.

## 4. Discussion

*DLK1* is crucial for the proper development of several mammalian tissues [38]. However, the specific functions and signaling mechanisms of *DLK1* in skeletal muscle cells remain unclear. We revealed that *DLK1* is expressed in a variety of tissues and highly expressed in the muscle tissue, reflecting the close association between *DLK1* and muscle development. This study was designed to elucidate the role of porcine *DLK1* in skeletal muscle development by overexpression of *DLK1*. Moore et al. [51] and Ohno et al. [52] showed that *DLK1* is preferentially expressed in fetal stromal cell lines and supports hematopoietic stem cell growth, directly demonstrating that exogenous *DLK1* is a positive regulator of murine stem cell growth. Stromal *DLK1* promotes proliferation of the intestinal epithelium during development [53]. Similarly, our data showed that mouse myoblast cells overexpressing porcine *DLK1* grew faster than the controls, and *DLK1* overexpression upregulated the expression of proliferation markers, thereby confirming a positive role of *DLK1* in myocyte proliferation. A study that upregulated *DLK1* in transfected K562 cells also enhanced myocyte proliferation, providing evidence for such a role [54]. However, the results of the present study are somewhat inconsistent with some previous results in terms of proliferation. Jolena et al. indicated that *DLK1* overexpression inhibited cell proliferation [36]. The lack of *DLK1* showed an enhanced number of cells in another study [55]. *DLK1* in hematopoietic cells inhibited proliferation and differentiation [56]. These functional differences may exist because different biological functions of *DLK1* are realized by alternative splicing of exon 5, and different subtypes seem to have different functions [57]. The function of *DLK1* may also be tissue specific. 

Muscle differentiation is an important process in skeletal muscle development. Skeletal muscle satellite cells differentiate into myoblasts, and *MyoD* and *MyHC* are important genetic indicators of this process. Several previous studies have indicated that *DLK1* may be involved in regulating cell differentiation [55,56,58]. Our data that the expression levels of *DLK1* gradually improved with the extension of differentiation time of C2C12 cells is consistent with the results observed by other investigators [36]. Compared with the control cells, both the number of differentiated myoducts and the expression of differentiated factors were increased in the *DLK1* gene overexpression group, which further proves the positive regulation of *DLK1* on cell differentiation. A new observation was reported in the current study. *DLK1* is associated not only with myocyte proliferation and differentiation, but also with cell migration. 

Myoblast fusion is a complex and highly regulated process and is one of the key steps in myogenesis [9]. Many genes and their products may regulate myoblast fusion, and their precise multilevel interactions are essential for myoblast fusion [59]. *Myomaker* and *β-1integrin* genes are mainly expressed on the surface of myoblasts and play an important role in regulating myoblast fusion [60]. In the current study, *DLK1* positively regulated myocyte fusion. Overexpression of *DLK1* led to more multinuclear fusions in differentiated cells, resulting in thicker and longer myotube formation and upregulated expression of the fusion markers, *Myomaker* and *β-1integrin*. Davis et al. [37] reported increased immunostaining of *DLK1* in callipyge LD skeletal muscle at 8 weeks of age, suggesting the potential role of *DLK1* in muscle hypertrophy. Our study showed that over-expressed *DLK1* significantly enhanced the expression level of muscle hypertrophy genes but reduced the expression of muscle degradation genes. These results are consistent with that in a previous report [21]. 

The signaling pathways that result in myogenesis are complex [61], and little is known about *DLK1* signaling in skeletal muscles. Notch has been widely studied as a key signaling pathway in skeletal muscle development in mice [62]. The expression of myogenic factors and the differentiation of multinucleated myotubes are suppressed by activating the Notch pathway [52,63,64]. The Notch signaling pathway has also been shown to regulate satellite cell activation, proliferation, differentiation, and muscle regeneration in mice [65]. Studies have suggested that *DLK1* is involved in the regulation of the Notch signaling pathway [66,67]. However, specific regulatory sites remain unclear. The findings of the current study support these reports, since *DLK1* inhibited Notch signaling by modulating *Hey1/2* and *Notch3* expression. Notably, *DLK1* did not affect *Notch1*. These results further demonstrate that *DLK1* acts as an inhibitor of the Notch pathway and regulates muscle development. 

Taken together, these observations suggest that *DLK1* has a positive regulatory effect on muscle growth and development, which may be mediated by the inhibition of the Notch signaling pathway. This finding provides a foundation for research on the mechanisms of muscle growth and development in pigs. *DLK1* may have many other functional roles that need to be explored; it remains to be determined whether there is a balance between the regulatory effects of different subtypes of *DLK1* on muscle development. In addition, the regulatory relationship between *DLK1* and the Notch pathway can be further verified by means of the specific activators of the Notch pathway, and it is necessary to clarify the form through which *DLK1* inhibits key loci of the Notch pathway.

## 5. Conclusions

We report the expression, functional identification, and regulatory pathways of porcine *DLK1*. *DLK1* is highly expressed in the muscle tissue of pigs. Cooperating cell phenotype and expression profile analyses showed that *DLK1* promoted myocyte proliferation, migration, differentiation, polynuclear fusion, and muscle hypertrophy, but inhibited muscle atrophy. In addition, *DLK1* also suppressed the Notch signaling pathway by regulating the expression of key factors in the pathway. These results lead to the conclusion that DLK1 might promote muscle growth and development by inhibiting the Notch signaling pathway, providing new insights and a foundation for further research on the molecular mechanisms underlying porcine myogenesis.

## Data Availability

Not applicable.

## Supplementary Materials

The following supporting information can be downloaded from https://www.mdpi.com/article/10.3390/ani12121523/s1: Table S1 Primer sequences for SqRT-PCR and qRT-PCR.
