# Supplementary material for: Functional Identification of Porcine DLK1 during Muscle Development
Source: Animals (Basel). 2022 Jun 11;12(12):1523. doi: 10.3390/ani12121523 (PMC9219491; doi:10.3390/ani12121523)
Supplement: Supplementary file 1 [file animals-12-01523-s001.zip › animals-1735100-supplementary.pdf]

Table S1. The primer sequences for SqRT-PCR and qRT-PCR.

Species	Genes	Primer Sequences (5′ to 3′)
Pig	*DLK1*	F: CGTCTTCCTCAACAAGTGCG<br>R: CTCCTCGTCCCCAGCCTC
	GAPDH	F: CACCATCTTCCAGGAGCGAG<br>R: CCCTTCAAGTGAGCCCCG
Mouse	*Ki67*	F: ATCATTGACCGCTCCTTTAGGT<br>R: GCTCGCCTTGATGGTTCCT
	Cyclin B	F: AATACCTACAGGGTCGTGAAGTGA<br>R: GCTGTATCATCTTCTTGGGCAC
	CDK4	F: GCTGCTACTGGAAATGCTGACC<br>R: AGCCTTGGGGGGAAACAGA
	p27	F: CAGGCAAACTCTGAGGACCG<br>R: TCGGGGAACCGTCTGAAAC
	BAD	F: GCTTAGCCCTTTTCGAGGAC<br>R: GATCCCACCAGGACTGGAT
	MyoD	F: CGAGCACTACAGTTGGCGACTAAGAT<br>R: GCTCCACTATGCTGGACAGGCAGT
	MyoG	F: CCATCCAGTACATTGAGCGCCTACA<br>R: ACGATGGACGTAAGGGAGTGCAGAT
	MyHC	F: CAAGTCATCGGTGTTTGTGG<br>R: TGTCGTACTTGGGCGGGTTC
	Myomaker	F: ATCGCTACCAAGAGGCGTT<br>R: CACAGCACAGACAAACCAGG
	β-1integrin	F: TTACAAGAGTGCCGTGACAACTG<br>R: GACTAAGATGCTGCTGCTGTGAG
	Atrogin1	F: GCAGCTGGATTGGAAGAAGA<br>R: GAGCAGCTCTCTGGGTTGTT
	Bmp4	F: CCGGATTACATGAGGGATCT<br>R: CCTGGGATGTTCTCCAGATG
	Murf	F: GCAAGGCTTTGAGAACATGG<br>R: TCTTCCTCATCAGCCTCCTC
	Foxo3	F: ACAAACGGCTCACTTTGTCC<br>R: CTGTGCAGGGACAGGTTGT
	Fst	F: TCTCTGCGATGAGCTGTGTC<br>R: CCTCCTCTTCCTCCGTTTCT
	Nog	F: TGTGGTCACAGACCTTCTGC<br>R: GTGAGGTGCACAGACTTGGA
	Hey1	F: GGAGGGAAAGGTTATTTTGACG<br>R: GCTGGGATGCGTAGTTGTTG
	Hey2	F: ATAGAAAAAAGGCGTCGGGA<br>R: CCTTTACCCCCTGTAGCCTG
	Notch1	F: GCCTTCGTGCTCCTGTTCTT<br>R: CCCACTCGTTCTGATTGTCG
	Notch3	F: TGAGAGTCTGATGGGGGAGG<br>R: GCCAGCATAAGTGGGGTGA
	GAPDH	F: TGCTGAGTATGTCGTGGAGTCT<br>R: ATGCATTGCTGACAATCTTGAG
